# Abnormal response to chronic social defeat stress and fear extinction in a mouse model of cholinergic dysregulation

**DOI:** 10.21203/rs.3.rs-2492514/v1

**Published:** 2023-01-30

**Authors:** Kristin R. Anderson, Peter J. Rogu, Talulla B. Palumbo, Julie M. Miwa

**Affiliations:** Lehigh University; Lehigh University; Lehigh University; Lehigh University

## Abstract

Cholinergic signaling is critical for an individual to react appropriately and adaptably to salient stimuli while navigating a complex environment. The cholinergic neurotransmitter system drives attention to salient stimuli, such as stressors, and aids in orchestrating the proper neural and behavioral response. Fine-tuned regulation of the cholinergic system has been linked to appropriate stress responses and subsequent mood regulation while dysregulation has been implicated in mood disorders. Among the multiple layers of regulation are cholinergic protein modulators. Here, we use validated models of experiential-based affective disorders to investigate differences in responses to stress in a genetic mouse model of cholinergic dysregulation based on the loss of protein modulator. The lynx2 nicotinic receptor modulatory protein provides negative cholinergic regulation within the amygdala, medial prefrontal cortex, and other brain regions. We discovered here that lynx2 knockout (KO) mice demonstrate an inability to update behavior with an inability to extinguish learned fear during a fear extinction test. We also observed, under an increased stress load following exposure to chronic social defeat stress (CSDS) paradigm, there was a unified resilience phenotype in lynx2KO mice, as opposed to the wild-type cohort which was split between resilience and susceptible phenotypes. Furthermore, we provide evidence for the functional role of α7 nicotinic receptor subtypes by phenotypic rescue with MLA or crossing with an α7 null mutant mouse (e.g. lynx2/α7 double KO mice). We demonstrate a direct physical interaction between lynx2 and α7 nAChR by co-immunoprecipitation of complexes from mouse BLA extracts. The genetic predisposition to heightened basal anxiety-like behavior and altered cholinergic signaling impairs individual behavior responses stressors. Together, these data indicate that the effects of social stress can be influenced by baseline genetic factors involved in anxiety regulation.

## Introduction

The stress response is an adaptive suite of physiological and psychological changes that allow an individual to galvanize appropriate responses to threatening situations (Schneiderman et al., 2005). If not regulated properly, however, individuals can develop persistent, pervasive, and generalized anxiety or fear in the form of an affective disorder. The capacity to constrain one’s response to stressors—to balance adaptive protection in the short term and maladaptive damage in the long term—is critical as disorders involving anxiety and fear are among the most prevalent mental disorders, affecting 33.7% of US adults at some point in their lives. (McEwen 2017; Kessler et al., 2012). Despite the high prevalence of affective disorders, current treatments do not meet the full need, highlighting the utility of further understanding the complex underpinnings of anxiety to inform the development of effective treatments.

Nicotinic acetylcholine receptors (nAChRs) of the cholinergic system have been shown to regulate activity in anxiety, fear, and depressive- related circuits; and related behaviors in animal studies (Picciotto 2003; Klein & Yakel, 2006; Gozzi et al., 2010; Mineur et al., 2016; Jiang et al., 2016; Wilson & Fadel 2017; Mineur & Picciotto 2019). Subsequently, the use of nicotine has been demonstrated to perturb affect in both directions, increase and decrease, in various contexts (Kutlu & Gould 2015; Mineur & Picciotto 2019). A cholinergic modulatory gene, lynx2, is expressed in key regions of anxiety and fear circuitry, such as the amygdala and medial prefrontal cortex, in addition to other CNS sites (Dessaud et al., 2006; Tekinay et al., 2009). The lynx2 protein binds to and reduces the activity of nAChRs (Tekinay et al., 2009). The lynx2 null mutant mouse (lynx2KO) displays heightened fear and anxiety-like behavior across several paradigms of basal anxiety. As the development of affective disorders often results from long-term adaptation to dynamic and complex conditions (such as basal anxiety and context), further elucidation of lynx2KO mice in paradigms that model these conditions can provide critical information (Erlich et al., 2012; Calhoun & Tye 2015; Anderson & Adolphs 2014).

A paradigm to study the updating of learned associations in humans and mice is fear extinction training (Lissek et al., 2004; Grillon 2008; Craske et al., 2014). Following fear conditioning, an associative learning process commonly implemented by a tone/shock pairing, fear extinction can be accessed by presentations of the tone without the shock in a new safety context. Extinction is an active learning process that produces a new safety memory, resulting in a scenario where the conditioned stimulus, the tone, no longer predicts the shock (Morgan et al., 1993; Herry et al., 2008; Furini et al., 2014). Fear extinction provides insight into the complex interactions of learning and basal states in a changing landscape.

Social stress is a pervasive form of stress experienced by most animals and is a known component of many human affective disorders such as depression and anxiety (Charney & Manji 2004; Krishnan et al., 2007). An etiologically relevant paradigm of conflict for the rodent species is the chronic social defeat stress test (CSDS), where test mice undergo daily defeat sessions characterized by both physical bouts and extended sensory contact with an aggressor mouse (Golden et al., 2011). CSDS is a highly validated assay that produces two divergent stress-responses in social interaction tests (Berton et al., 2006; Lagace et al., 2010; Golden et al., 2011; Hultman et al., 2018).

We hypothesized that the dysregulation of nAChRs in neuronal circuits regulating affective outputs by lynx2 removal, and subsequent heightened basal anxiety would alter these complex behaviors (Dessaud et al., 2006; Tekinay et al., 2009). We predicted that lynx2KO mice would display a maintenance of fear during the fear extinction paradigm and would be disproportionately affected after defeat, displaying increased anxiety-like and fear-related behaviors. Here, we show that lynx2KO mice display a robust lack of fear extinction, but contrary to our prediction, the lynx2KO mice switched from socially avoidant before defeat to a social approach/resilient phenotype after defeat. The differential responses, both in the initial response and long-term perturbations, to stressors may underlie the likelihood of developing a disorder and provide potential screening tools to identify high risk individuals.

## Methods And Materials

### Animal Model.

All animal procedures were conducted in accordance within the guidelines of Lehigh University IACUC. Animals were kept on a 12/12 light dark cycle with ad libitum access to food and water. Animals were weaned at 21 days of age and separated by sex into group housing ranging from 2–5 animals per cage. 3–8-month-old naïve, male and female, wildtype (WT) (C57BL/6J), lynx2KO, and double null mutant mice for the alpha7 nAChR and lynx2 (alpha7-lynx2KO mice) were used. Breeding of the lynx2KO mice included null mutant crosses to create knockout mice for experiments, backcrosses the C57BL/6J mice to maintain genetic diversity and avoid genetic drifts, and crosses of heterozygous mice from the backcrosses to other heterozygous mice from different pairs or knockout mice to refresh to the null mutants. The backcross was introduced every three generations. The alpha7- lynx2KO mice were created by crossing lynx2KO null mutant mice with alpha7 null mutant mice. Litters from first generation double heterozygous mice were crossed together and second generation mice were genotyped to find either one allele as a null mutant and the other allele as heterozygous or for double mutants. Combinations of the possible genotypes (such as lynx2KO, alpha7 heterozygous and lynx2 heterozygous and alpha7KO) were crossed until several double mutants were verified. The double mutants were crossed to create mice for experiments. Backcross to C57BL/6J mice were added every 3 generations and added into the breeders as described before to avoid genetic drift. All mice were genotyped using a polymerase chain reaction assay from DNA that was extracted from a tail biopsy. Each tissue sample was genotyped 2–3 times before a confirmation of the genotype was assigned. All mice were involved in one behavioral assay.

### Statistics and Graphs.

Power analysis was conducted before data collection. For each experiment, an ANOVA was run to compare output (freezing or latency to dark) to age and sex. When no significant differences were obtained for age or sex, all ages and sexes were combined to analyze by genotype. Statistical analyses between groups were performed using a two-tailed t-test, with a p-value less than 0.05 considered to be statistically significant, by a repeated one-way ANOVA reporting interaction (such as genotype*time) Wilks’s Lambda values followed by either paired T-test or Bonferroni post hoc tests where appropriate, or by a Two-way ANOVA. A Cohen’s d test was used to determine effect size. All behavioral assays were performed with a minimum of 3 replicates or independent cohorts to ensure a biological phenomena as opposed to a one time result. Replicates were temporally separated tests and each included a different set of wildtype and knockout mice. All data are given as 95% confidence intervals (CI) and are represented as AVG ±SEM. *p<0.05, **p<0.01, ***p<0.001.

### Fear Conditioning and Extinction

Fear conditioning was carried out in a conditioning hub (Coulbourn Instruments) placed inside an isolation cubicle (Coulbourn Instruments) to prevent ambient light and sound interference. The hub environment was lined with silver metallic walls, washed with isopropyl alcohol between trials, and had a mounted shock floor. On day 1, mice underwent a 2-minute acclimation followed by 2, 30 second sound (80 dB and 8kHz) presentations 2 minutes apart. To induce fear conditioning, during the last 2 seconds of the sound the animal received a mild (0.5 mA) foot shock. Thirty seconds after the last foot shock the animals were returned to their home cage. For the weaker fear extinction paradigm, mice received only 1 tone-shock pairing and a 0.25 mA foot shock. This was run with a different set of mice than the 2 tone-shock pairing. On day 2 and 3 fear extinction took place, with multiple measures taken to control for context. Mice were placed in a different cubicle than the one used for conditioning, the walls of the hub were switched to colorful patterns, a lavender scent was introduced, and between trials, the hub was washed with the novel cleaning agent, ethanol. After 30 seconds of acclimation in the extinction chamber, the animal was presented with 3 sound presentations at the same frequency and intensity as on the training day, each 30 seconds long and 2 minutes apart, but with no foot shocks.

The behavior of the mice was recorded using an in-hub camera and time spent freezing was analyzed using Coulbourn FreezeFrame software. Freezing was considered to be no bout of movement in a 2 second frame. The criteria for an extinguished fear response was a statistically significant decrease in percent freezing to tone (CS) between day 2 and day 3.

### Fear Conditioning and Extinction with pharmacological treatment

Fear conditioning was performed as previously described, but with the addition of an IP injection given to lynx2KO mice and WT mice 1 hour prior to fear extinction on day 2 and 3. Mec (a nonspecific nAChR blocker), MLA (an α7 antagonist), and DHβE (a β2* antagonist) can cross the blood brain barrier at several documented doses, with enough potency to produce changes to nAChR function (Turek et al., 1995; Damaj et al., 1995). Doses were chosen based upon such studies. Mec (0.3–2.5 mg/kg, Abcam, Cambridge, MA), DHβE (2 mg/kg, Tocris Bioscience, Bristol, UK), and MLA (5 mg/kg, Sigma – Aldrich, St Louis, MA) were dissolved in 0.9% saline. The control animals were injected with 0.9% saline. Successful fear extinction was defined as a decrease in percent freezing from day 1 to day 2. Day 2 will be analyzed as the final extinction time point.

### Light Dark Box

The light dark box assay was conducted in TruScan motion tracking arena (Coulbourn Instruments). A dark walled box was placed inside the back half of the arena with the front half surrounded by clear walls. An arched opening was made between the two areas. lynx2KO or WT mice were initially placed in the light side and their location was monitored for 10 minutes by the TruScan software. Increased latency to enter the dark and increased time spent in the dark indicates heightened anxiety-like behavior. For pharmacological studies, mice were given an. injection, IP, 1 hour prior to the start of testing.

### Chronic Social Defeat Stress

CSDS followed a method adapted from the standardized protocol (Golden et. al., 2011). First, CD1 mice were singly housed for 7 days followed by a screen for aggression over 3 days. During each day of screening, an 8–20-week-old WT male was added to the home cage of the CD1 and the latency of the CD1 to attack is recorded. The WT mouse was removed upon attack or after 3 minutes. CD1 aggressors must show aggression in at least 2 subsequent sessions and have an attack latency of less than 60 seconds to be considered as an aggressor.

For non-defeated mice (WT, lynx2KO, a7L2KO) were housed with litter mates after weaning. They were placed in the same room as defeated mice and not disturbed until they were used for social interaction. For defeat, test mice (WT, lynx2KO, α7L2KO) were exposed to a CD1 aggressor mouse for a daily bout of 10 attacks. Compared to C57 (WT), CD1 mice display more aggression than C57 mice (Hsieh et al., 2017). An attack was defined as a physical altercation that included, but was not limited to, biting, shoving, rushing, jumping onto, tail rattling, and kicks. The attacks were limited to 10 to prevent excessive harm to the test mice. Following the bout, the intruder mouse was physically separated from the CD1 aggressor within the CD1 home cage but kept in sensory contact for 24 hours. This was repeated each day for 10 days, after which, the test mice were singly housed for 24 hours to await behavioral testing.

### Modified Chronic Social Defeat

We adapted the 10-day CSDS to test the robustness and sensitivity of the post-defeat phenotypes. The same procedure as above was conducted but the defeat sessions occurred over 3 days. After the 3rd day of defeat the test mice were singly housed. We found that the 3 days was sufficient to induce enough of a response in the test mice that conferred with the matching behavioral results of the 10-day protocol.

### Social Interaction

The social interaction (SI) test occurred 24 hours after the defeat session ended and mice were singly housed. SI ratios were assessed in Coulbourn TruScan motion tracking arenas. The arena was calibrated with an interaction zone against the back-most wall and a wire containment cage was built to the specifications of the protocol (Golden et al., 2011).

For each phase of social interaction test, post-defeat mice or controls (naïve WT and L2KO mice that did not undergo defeat and were kept in the standard housing of 2–5 littermates), called defeated or non-defeated test mice respectively, were placed in the center arena and tracked for 150 seconds. The initial phase consisted of an empty interaction zone cage. Immediately following the completion of phase 1, the test mouse was removed from the arena and returned to its home cage. During the break, a novel CD1 stranger mouse was added to the wire containment cage within the interaction zone. The same test mouse was again placed in the center of the arena and tracked for 150 seconds. During this second phase, sensory information could be transmitted from the stranger to the test mouse but there was no physical contact.

The location of the test mouse was tracked by the TruScan software. Social interaction ratios were determined based on time in the target zone (24 × 14 × 9 cm box surrounding the interaction zone) with and without the stranger mouse present. The social interaction (SI) ratio was used to assign the test mouse to a group: resilient or susceptible. The SI ratio is calculated by the following formula:

$$SI=\frac{\text{Time}\;\text{spent}\;in\;\text{interaction}\;\text{zone},\text{stranger}\;\text{present}}{\text{Time}\;\text{spent}\;in\;\text{interaction}\;\text{zone},\text{empty}\;\text{zone}}$$

For these studies, an SI ratio above 1.5 was considered ‘resilient’ and below 1.5 ‘susceptible’ rather than the standard 1 as the lynx2KO mice were shifted towards higher SI ratios. We choose an SI ratio of 1.5 as our cutoff to increase the criteria needed to reach the resilient phenotype. Upon separating the groups, the data are presented as the percentage of time spent in the interaction zone with the stranger mouse present.

Test mice first underwent a CD1 social interaction test with a CD1 stranger mouse on day 11. The stranger mice used in the social interaction were either naïve to the test mouse or in some cases for the CD1, the stranger was the most remote CD1 aggressor to that test mouse, from day 1 of CSDS. Only males were used in these studies due to the di culty of initiating attack behavior directed toward female mice (Takahashi et al., 2017).

### Co-Immunoprecipitation (Co-IP)

**WT**, **lynx2KO, and alpha7KO mice were anesthetized with isoflurane and rapidly decapitated. The BLA was bilaterally dissected using visual landmarks** and immediately homogenized in the bullet blender tissue homogenizer (NextAdvance, Averill Park, NY, USA), with Co-IP buffer (50 mM Tris, 150 mM NaCl, 0.75% Triton-X 100, Pierce protease inhibitor cocktail). Dynabeads A (ThermoFisher Scientific,Waltham, MA, USA) were pre-incubated with 5μg rabbit anti-lypd1 (lynx2) primary antibodies (ThermoFisher Scientific, Waltham, MA, USA) and thoroughly washed. An input sample of each homogenate was removed and kept at −80 degrees celsius until western blot analysis. Brain homogenates were incubated at a concentration of 13 mg/ml with the Dynabeads-antibody complex for 3 days nutating at RT. After washing, lynx2 (lypd1) protein complexes including interacting proteins were eluted and immediately prepared for western blot analysis. Input (sample prior to the immunoprecipitation) and supernatant (after the immunoprecipitation) lane were to be compared to IP samples in the western blot analysis. Co-immunoprecipitation experiments were performed in three replicates, each with 1 lynx2KO mouse and 2 WT mice for a total of 18 technical replicates with 9 animals. The lynx2KO mice were used as a negative control for the lypd1 antibody and the alpha7KO mice were used as a negative control for the alpha7 antibody.

### Western Blot analyses

Samples were denatured in 1x sample buffer (ThermoFisher Scientific,Waltham, MA, USA) at 95°C and run on a 4–20% gradient SDS-PAGE gel (Bio-Rad Laboratories, Hercules, CA, USA). Gels were transferred onto activated PVDF membrane via a semi-dry transfer. The membrane was blocked with 5% milk/0.05% Tween-PBS for 1 hour at 4°C followed by an incubation overnight at 4°C in mouse monoclonal anti-nicotinic acetylcholine receptor α7 Subunit (1:1000, Sigma-Aldrich, St Louis, MO) or rabbit polyclonal anti-BDNF (5 μg/mL, Millipore, Burlington, MA, USA) for the BDNF study. After thorough washing, membrane was incubated with conjugated goat anti-mouse (Abcam, Cambridge, MA, USA) at 1:10,000 for 2 hours at 4°C or 1:10,000 or conjugated goat anti-rabbit (Abcam, Cambridge, MA, USA) at 1:10,000 for 2 hours at 4°C for BDNF study. Membranes were incubated in ECL (ThermoFisher Scientific,Waltham, MA, USA) and exposed to film. Loading controls were run using mouse anti-actin primary antibodies (Abcam, Cambridge, MA, USA) at 1:1000 dilution, and goat anti-mouse secondary antibodies (Life Technologies, Carlsbad, CA, USA) at 1:40,000 dilution.

## Results

### The loss of lynx2, in a lynx2KO mouse, leads to a robust lack of fear extinction

**Figure 1:**
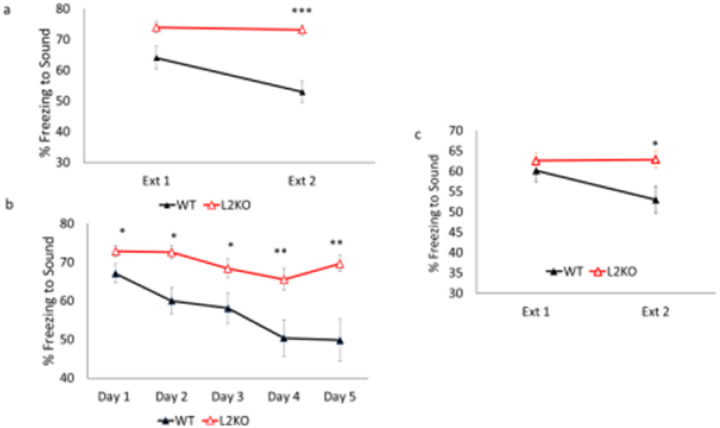
Marked lack of fear extinction in lynx2KO mice a) Over the course of fear extinction, lynx2KO mice (red line) do not undergo fear extinction asdemonstrated by the maintenance of freezing. WT mice (black line) do undergo fear extinction and decrease freezing over the course of extinction. Two-way ANOVA for gender*genotype F=0.743, p=0.395. As there is no difference between male and females, sexes are combined. A one-way repeated measures ANOVA was conducted to compare the effect of genotype on freezing during extinction day 1 and extinction day 2. There was a significant effect of genotype; Wilks’s Lambda = 0.766, F (1, 34) = 7.918, p=0.011. Two paired T-tests were used for post hoc comparisons. A first paired samples t-test indicated a significant effect of genotype on day 1 freezing; t (35)=35.07, p<0.00. A second paired samples t-test indicated a significant effect of genotype on day 2 freezing; t(35)=32.757, p<000. WT n =20, L2KO n=19, WT 95% CI [51.548, 64.224], L2KO 95% CI [69.536, 76.310], Cohen’s d = 1.535 b) In an extended fear extinction protocol, WT mice (black line) display a reduction in percent freezing totone/CS while lynx2KO mice (red line) have an overall maintenance of fear. Although lynx2KO mice show a trend in inter-trial (within extinction day) extinction after several sound presentations, the reduction in freezing from day to day is not significant resulting in an overall maintenance of freezing and lack of extinction behavior. A one-way repeated measures ANOVA was conducted to compare the effect of genotype on freezing during extinction day 1 thru extinction day 5. There was a significant effect of genotype; Wilks’s Lambda = 0.410, F (4, 24) = 4.654, p=0.006. Five paired T-tests were used for post hoc comparisons. A first paired samples t-test indicated a significant effect of genotype on day 1 freezing; t (28)= 50.919, p<0.00. A second paired samples t-test indicated a significant effect of genotype on day 2 freezing; t(28)= 33.236, p<0.00. A third paired t-test indicated a significant effect of genotype on day 3 freezing; t(28) = 28.178, p<0.00. A fourth paired t-test indicated a significant effect of genotype on day 4 freezing; t(28) = 21.075, p<0.00. A fifth paired t-test indicated a significant effect of genotype on day 5 freezing; t(28) = 20.199, p<0.00 WT n=12, L2KO n = 16. Day 5 95% CI: WT[53.230, 60.804], KO[67.828, 71.665], Cohen’s d= 1.089. c) With a weaker training there are no differences in fear learning, as demonstrated by percent freezing to tone (CS) on day 1, but the lynx2KO mice (red line) exhibit a lack of fear extinction behavior as compared to WT mice (black line). A one-way repeated measures ANOVA was conducted to compare the effect of genotype on freezing during extinction day 1 and extinction day 2. There was a significant effect of genotype; Wilks’s Lambda = 0.706, F (1, 17) = 7.095, p=0.016. Two paired T-tests were used for post hoc comparisons. A first paired samples t-test indicated no significant effect of genotype on day 1 freezing; t (18)=31.983, p=0.542. A second paired samples t-test indicated a significant effect of genotype on day 2 freezing; t(18)=24.055, p<0.00. WT n=10, L2KO n=10, WT 95% CI[44.074, 61.840], L2KO 95% CI [57.740, 67.904], Cohen’s d = 0.996.

To discern how lynx2KO mice responded in a paradigm that requires the updating of previously learned associations in response to new information, we tested the lynx2KO in a fear extinction paradigm. Mice were subjected to two pairings of an innocuous tone and a foot shock, and 24 hours later, were tested for freezing behavior in response to the tone alone (CS), presented over 2 days, each consisting of three CS trials per day. We found that lynx2KO mice displayed heightened fear responses and a lack of fear extinction, as demonstrated by a maintained level of freezing over the course of extinction trials (Fig1a). To establish the strength and longevity of the abnormal response in the fear extinction paradigm of lynx2KO mice, we extended the number of fear extinction sessions beyond six trials over two days. Fear extinction is an active process that results in the formation of a new memory in which the CS is no longer predictive of the US. As such, it is possible the neurons of the lynx2KO mice need additional input to form a stable safety memory that can outcompete the original fear memory. To determine if the lynx2KO mice lack the capacity to undergo extinction or if the underlying cellular processes need more input to induce extinction behavior, a longer-term fear extinction protocol was utilized in which the tone/CS was presented three times a day for a total of five days. Over the course of five days, lynx2KO continue to display a lack of extinction behavior compared to WT mice (Fig1b). A possible confound in assessing extinction ability could be the heightened fear learning exhibited by lynx2KO mice. To mitigate this, a milder training protocol was implemented prior to the fear extinction test, using a single tone-shock pairing and reduced shock intensity to develop the association. The heightened fear learning in lynx2KO mice was lost in this paradigm, and yet the WT and lynx2KO mice exhibited the same level of freezing on the first extinction trial, and under these conditions, the lynx2KO mice maintain the freezing behavior over the course of extinction, indicating that they have abnormal fear extinction capability (Fig1c). No differences in sex or age were found (Supplemental Figure #)

### nAChR blockade and antagonism rescues the lack of fear extinction, implicating the α7 nAChR subtype in fear extinction

**Figure 2:**
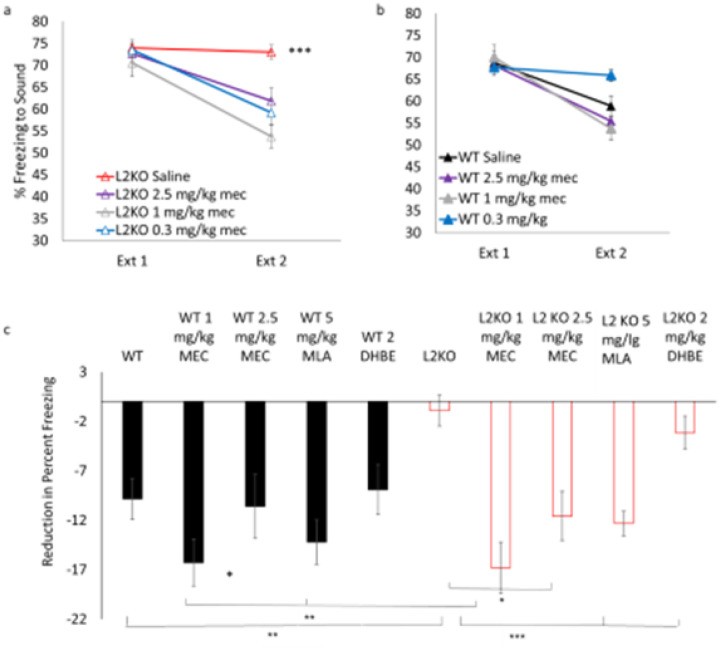
Cholinergic blockade implicates distinct nAChRs in lynx2-mediated behaviors a) Mecamylamine, at all doses tested, rescues the lack of fear extinction in lynx2KO mice by restoring a reduction in percent freezing to tone (CS) as compared to lynx2KO mice without mecamylamine (red line). b) These doses have no effect in the WT mouse in this paradigm (FigS1a). A one-way repeated measuresANOVA was conducted to compare the effect of the drug in lynx2KO mice on freezing during extinction day 1 and extinction day 2. There was a significant effect of drug; Wilks’s Lambda = 0.750, F (1, 56) = 6.236, p=0.001. Bonferroni post hoc; L2KO vs L2KO 0.3 mg/kg (M=−7.154, SE=2.568) p= 0.044,L2KO vs L2KO 1 mg/kg (M=11.337, SE=2.66) p< 0.001,L2KO saline n=20, L2KO 2.5 mg/kg n=11, L2KO 1 mg/kg n=14, L2KO 0.3 mg/kg n= 10. 95% CI, L2KO[69.536,76.310], L2KO 2.5 mg/kg [53.717, 69.934], L2KO 1 mg/kg [47.831, 59.615], L2KO 0.3 mg/kg [53.184, 65.183]. c) α7, not α4β2, nicotinic receptor antagonism restores extinction behavior in the lynx2KO mouse as demonstrated by a reduction in percent freezing to tone (CS) (negative value) over the course of extinction trials. Black bars represent WT mice and white bars represent lynx2KO mice. One-way ANOVA F (7) = 10.629, p<0.001. WT saline n= 16, L2KO saline n=20, WT 1 mg/kg mec n=17, WT 5 mg/kg MLA n=11, WT 2 mg/kg DHBE n=10, L2KO 1 mg/kg mec n=14, L2KO 5 mg/kg MLA n=14, L2KO 2 mg/kg DHBE n=15, 95% CI: WT [51.548, 64.244], L2KO [69.536,76.310], WT 1 mg/kg mec [47.952, 59.531], L2KO 1 mg/kg mec[47.831, 59.615], WT 5 mg/kg MLA[49.543, 60.731], L2KO 5mg/kg MLA [57.257, 62.779], WT 2 mg/kg DHBE [57.653,69.174], L2KO 2 mg/kg DHBE [65.121,75.704].

We tested the ability of nAChR pharmacological treatment to restore normal extinction behavior in the lynx2KO. Injection of mecamylamine, a general nAChR blocker, rescued fear extinction behavior in the lynx2KO mice at all doses tested (Fig2a). Utilizing more specific nAChR antagonists, we found that methyllycaconitine (MLA), an α7 nAChR antagonist, prior to extinction rescued the lynx2KO fear extinction phenotype while dihydro-β-erythroidine (DhβE), a heteromeric nAChR antagonist, did not, suggesting that the lynx2-based extinction phenotype is α7 nAChR based (Drasdo et al 1992; Damaj et al 1995) (Fig2c). There were no significant responses to pharmacological manipulation in fear extinction for WT mice, suggesting a differential responsiveness, either in sensitivity or selectivity, of nAChRs in this paradigm when lynx2 is present (Fig 2b–c). We have further demonstrated that the pharmacological manipulation of nAChRs through the same agents used in fear extinction studies mecamylamine and MLA, rescued a known phenotype in lynx2KO mice: increased anxiety-like behavior in the light-dark box assay (Tekinay et al., 2009). Injection of either the nAChR blocker mecamylamine and antagonist MLA rescued the elevated basal anxiety-like behavior observed in lynx2KO mice in the light dark box assay (FigS1a). Additionally, DHβE injection, which has no effect in lynx2KO mice in fear extinction behavior, could restore the behavior in the light dark box assay, demonstrating that DHβE at this dose can affect the lynx2KO mice but that it is likely different neurons with different nAChRs in possibly different neural circuits are recruited or activated to these two stimuli and underlie the two behaviors (FigS1a).

### lynx2KO mice display a population shift from socially avoidant to social resiliency behavior following defeat

**Figure 3:**
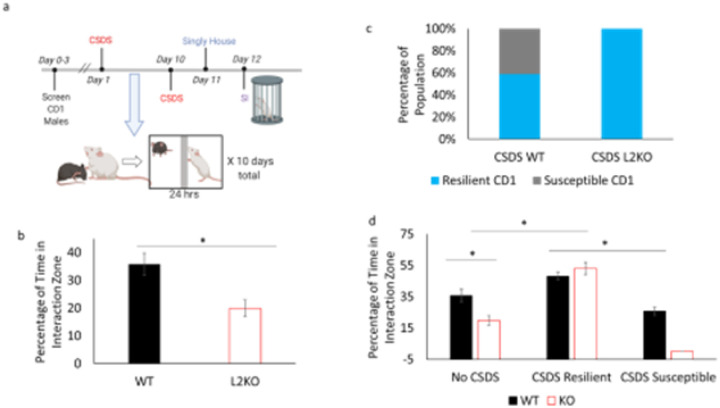
Shift in all social interaction behavior following defeat in lynx2KO mice a) Schematic of chronic social defeat stress timeline: prior to CSDS, CD1 mice are screened for aggression. Mice undergo 10 days of CSDS (chronic social defeat stress), entailed daily bouts of aggressive interactions with extended sensory contact. Following the 10^th^ day, mice are singly housed for 1 day before undergoing SI (social interaction). b) Prior to defeat, lynx2KO display social avoidance as demonstrated by a decreased percentage of time in the interaction zone. Test mice are placed chamber with a C57 mouse as the stranger mouse in this social interaction test. Student’s Two-Tailed T-Test p=0.004. WT n=17, L2KO n=15. 95% CI: WT[27.383, 44.147]. L2KO[12.504, 26.060], cohen’s d =1.118. c) After experiencing defeat stress, WT mice show a population split in social interaction phenotype between resilient (blue) and susceptible (gray) phenotypes in response to a CD1 mouse while lynx2KO mice exhibit a population phenotype of only resilient. d) Prior to defeat, lynx2KO mice display less time in the social interaction zone and exhibit social avoidance (No CSDS groups). Based upon social interaction ratios post-defeat, there are WT mice that approach and spend a greater percentage of time in the interaction zone (CSDS Resilient) as compared to WT mice that do not approach and spend less time in the interaction zone (CSDS Susceptible). There are no lynx2KO mice that display a non-approach/susceptible phenotype. All lynx2KO mice post-defeat spend a larger percentage of time in the interaction zone. One-Way ANOVA F=14.016, p<0.001, Bonferroni Post-hoc: WT vs L2KO p=0.019, WT vs CSDS L2KO p=0.005, L2KO vs CSDS KO p<0.001, L2KO vs CSDS WT Resilient p<0.001, CSDS WT Resilient vs CSDS WT Susceptible p=0.016, CSDS L2KO vs CSDS WT Susceptible p<0.001. WT n=17, L2 KO n=17, CSDS L2KO n=17, CSDS WT Resilient n=10, CSDS WT Susceptible n=7. 95% CI: WT [27.383, 44.147], L2KO[12.504, 26.060], CSDS Resilient WT [24.782, 44.551], CSDS Susceptible WT [27.017, 43.841], CSDS Resilient L2KO [44.495, 61.701]. Cohen’s d WT No CSDS vs KO NO CSDS=1.118, No CSDS L2KO vs CSDS Approach L2KO = 1.927.

To investigate how an etiologically relevant stressor impacted responses of mice predisposed to anxiety, we conducted a chronic social defeat stress (CSDS) paradigm followed by social interaction (Fig3a). We compared pre-defeat social interaction to post-defeat social interaction results. In social interaction tests, a test mouse is added to an arena with a restrained stranger mouse which allows for the flow of sensory information of the stranger without physical contact. The evaluation of the phenotype was conducted by generating an SI (social interaction) ratio of time spent in the interaction zone with the stranger mouse present vs when there was no stranger mouse present, using a cutoff of 1.5 (see methods). Prior to CSDS, lynx2KO were tested for baseline sociability levels in a standard social interaction test, where a conspecific C57 mouse is used as the stranger. The tested mice displayed expected social avoidance (Fig3b), consistent with previous reports (Tekinay et al., 2009). Thereby confirming the effective phenotype while allowing the CD1 mice to remain novel until the start of the CSDS sessions.

Following the completion of the CSDS paradigm, mice were tested in a social interaction test with a novel CD1 aggressor mouse as the stranger mouse. In this social interaction test, with the CD1 aggressor stranger mouse, defeated WT test mice displayed the expected split with divergent stress responses of the population, with individuals displaying either the resilient and susceptible phenotypes. On the other hand, all the defeated KO mice, even with an SI ratio cutoff that increases the criteria for resiliency, displayed the resilient phenotype (Fig3c). There was no significant difference in the time spent in the interaction zone by the resilient subset of WT mice and the lynx2KO mice. There were significant differences in the time spent in the interaction zone between the resilient mice and the susceptible WT mice (Fig3d).

### Removal of α7 rescues the L2KO phenotype in both fear extinction and chronic social defeat stress

**Figure 4:**
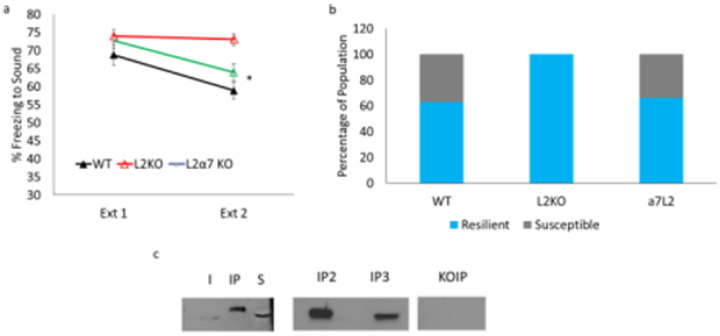
lynx2-mediated action though α7 nAChRs a) The double knockout (green line) with a deletion of both the α7 nAChR and lynx2 genes restores fear extinction behavior to that of WT levels (black line) as compared to lynx2KO mice (red line). A one-way repeated measures ANOVA was conducted to compare the effect of the genotype on freezing during extinction day 1 and extinction day 2. There was a significant effect of the genotype on freezing; Wilks’s Lambda = 0.836, F (1, 49) = 4.793, p=0.013. Bonferroni post hoc: WT vs L2 KO (M=9.6752, SE = 3.095) p=0.009, WT vs a7L2 (M=4.4857, SE=3.22) p=0.513, L2KO vs a7L2KO (M=8.3485, SE= 3.14) p=0.032. WT n=16, L2KO n=20, a7L2 n=16. 95% CI: WT[51.548, 64.244], L2KO[69.536,76.310], a7L2[58.846, 69.045]. Cohen’s d L2KO vs a7L2 = 1.050. b) In a 3-day CSDS paradigm, the WT and lynx2KO mice continue to display the same trends of the 10-day paradigm: WT mice display a divergent response while the lynx2KO mice display one population response. The double knockout restores the population response to that of WT levels with instances of both the susceptible and resilient phenotypes. c) Co-immunoprecipitation via lynx2 pull down and an α7 probe in a western blot analysis. α7 is enriched in all samples after immunoprecipitation (IP) as compared to Input (I) or Supernatant (S). IP2 and IP3 represent additional replicates from different biological samples prepared from different animals. No bands seen in the KO IP where the lynx2 pull down was performed in a lynx2KO mouse.

To confirm the pharmacological studies implicating α7 nAChRs in the fear extinction phenotype of lynx2KO mice, we sought a genetic confirmation of α7 nAChRs involvement. The fear extinction phenotype of lynx2KO mice was rescued by crossing the lynx2KO mice to α7 nAChR null mutant mice (α7KO), adding to the evidence that lynx2-based extinction is α7-based (Fig4a). In a 3-day CSDS, the α7lynx2 double knockout mice also displayed divergent stress responses similar to that of WT mice (Fig4b), supporting the involvement of α7 nAChRs in both fear extinction and CSDS paradigms. In the 3-day CSDS paradigm, lynx2KO mice also continued to display a resilient phenotype 100% of the time (Fig4b).

A lynx2 protein-nAChR complex has never been detected in vivo but is a critical condition for the functional relevance of the pharmacological and genetic results. To establish the physical interaction of lynx2 and α7 nAChR proteins in the brain co-immunoprecipitation experiments were performed to isolate lynx2-nAChR stable complexes in the mouse brain. To detect specific components of the complex, we immunoprecipitated the lynx2 protein from homogenates of the mouse amygdala and probed for the α7 nAChRs subunit using an anti-α7 nAChR antibody in Western blot analyses. Precipitated lynx2 complexes from extracts of WT brain samples yielded a band corresponding to the α7 nAChRs subunit, providing evidence that lynx2 forms a stable complex with α7 nAChR in the mammalian brain (Fig4c). No bands were seen in control samples prepared from lynx2KO mice, indicating the specificity of the interaction. Taken together, our results indicate the role of lynx2 modulation of α7 nAChRs in the modulation of fear extinction and CSDS behaviors.

## Discussion

This work explores the basic science of a genetic factor implicated in anxiety-like phenotypes, the lynx2 gene, and the involvement of lynx2-based cholinergic modulation of fear extinction and CSDS behaviors. In this study, we identified a specific and critical role for lynx2-mediated suppression of α7 nAChRs responses in both fear extinction and CSDS paradigms, which can be ameliorated through pharmacological or genetic perturbations of α7 nAChRs. Together with biochemical studies indicating a stable direct interaction of lynx2 and α7 nAChRs in the amygdala, these data indicate that lynx2- α7 complexes play an important regulatory role on cholinergic responsiveness during such complex behaviors

The data from this study indicate different profiles in the response to stressors, pointing towards a role of a fine-tuned cholinergic system in regulating behavioral responses. Mice null for lynx2 demonstrated both a marked lack of fear extinction and a unified, homogenous phenotype of resiliency following CSDS. WT mice, on the other hand, exhibit a more varied set of responses. Similar phenotypes have been demonstrated previously, as described in Meduri et al., 2013, between social interaction following defeat and fear extinction behaviors where resilient wildtype mice also displayed a lack of fear extinction. The mechanisms that result in both these phenotypes in wildtype and KO mice may be different but could also point to an important regulatory role of α7 nAChRs within key circuits underlying these behaviors as both fear extinction and CSDS phenotypes in the lynx2KO mouse could be ameliorated by antagonism of the α7 nAChR. Altered lynx2-mediated suppression of this nAChR subtype, both full loss in the KO or perturbed regulation in others that increases cholinergic signaling, may be the underlying cause of these perturbed phenotypes.

An alternative possibility is the role of plasticity mechanisms, which have been highly implicated in fear learning/extinction (Hascoët et al 2001; Jiang et al 2016; Jeongyeon et al 2007; Dalton et al 2012; Maren 2015; Saha et al 2017; Zhu et al 2017) and in responses to CSDS (Jansow et al., 2004; Jansow et al., 2005). Recent work has demonstrated that increased cholinergic singling, via exposure to nicotine, in wildtype mice enhances resiliency by inducing brain derived neurotrophic factor (BDNF), a protein implicated in many neuroplasticity processes, signaling in the hippocampus (Khalifed et al., 2020). Other work has linked increased BDNF in the VTA to susceptible defeat outcomes (Wook Koo et al., 2016). It is plausible that similar excitability increases that induce BDNF are present in other brain regions of the lynx2KO mouse due to activity dependent acetylcholine release (Acquas et al., 1996; Letzkus et al., 2011). We assessed BDNF levels following defeat in lynx2KO VTA and found a decreased level relative to controls. These data are consistent with the role of VTA BDNF in susceptibility (SupFig1b). Future work can further explore this avenue further to define plausible altered plasticity in the lynx2KO mouse as consequence of increased cholinergic signaling in specific brain regions.

The lynx2KO mouse model provides a framework for the development of a novel pharmacogenetic approach to understanding affective disorders. Genetic factors which predispose an individual to heightened basal anxiety-like behavior could impair appropriate responses to changing environmental conditions and to stressors. The differential responses in our genetic model, both in the initial basal response and long-term perturbations, to stressors raise a possibility that such alterations may underlie the likelihood of developing a disorder.

A genetic predisposition to increased fear-related behaviors, conferred by the loss of lynx2 and heightened cholinergic signaling, may play a role in the response to persistent social stress. In humans, individuals do vary in their responses to social stress and later vulnerability to psychiatric disorders (Charney & Manji 2004; Krishnan et al., 2007). Ultimately, defining the biological factors that underlie predispositions towards anxiety and how environmental stressors act to modify these biological factors can aid in the development of effective target-based therapeutics.

Our data suggest a possible screening tool and pharmacogenetic treatment direction. nAChR antagonism should be explored as a fruitful avenue for individuals harboring a deleterious lynx2 mutation (gnomAD, ExAC, and Bravo databases, Monkol et al., 2016) since mecamylamine, a general nAChR blocker used in this study, is a prescribed medication. Pharmacological intervention paired with behavioral training could ameliorate extinction of learned associations that are no longer adaptive in a specific context. These results have implications for the treatment of inextinguishable fear, such as that in post-traumatic stress disorder, phobias, and other severe disorders, and therefore could lay the groundwork towards optimized personalized therapies.

## References

[R1] Acquas E, Wilson C, Fibiger HC (1996) Conditioned and Unconditioned Stimuli Increase Frontal Cortical and Hippocampal Acetylcholine Release: Effects of Novelty, Habituation, and Fear. J Neurosci. 76(9): 3089–3096. 10.1523/JNEUROSCI.16-09-03089.1996 PMC6579062 8622138

[R2] Anacker C, Scholz J, O’Donnell KJ, Allemang-Grand R, Diorio J, Bagot RC, Nestler EJ, Hen R, Lerch JP, Meaney MP (2016) Neuroanatomic Differences Associated With Stress Susceptibility and Resilience. Biol Psychiatry 79(10): 840–849 26422005 10.1016/j.biopsych.2015.08.009 PMC5885767

[R3] Anderson DJ and Adolphs R (2014) A framework for studying emotions across species. Cell. 2014 Mar 27;157(1):187–200.: DOI: 10.1016/j.cell.2014.03.003 24679535 PMC4098837

[R4] Bagot RC, Cates HM, Purushothaman I, Lorsch ZS, Walker DM, Wang J, Huang X, Schlüter OM, Maze I, Peña CJ, Heller EA, Issler O, Wang M, Song W, Stein JL, Liu X, Doyle MA, Scobie KN, Sun HS, Neve RL (2016) Circuit-wide Transcriptional Profiling Reveals Brain Region-Specific Gene Networks Regulating Depression Susceptibility. Neuron 90 (5): 969–983. 27181059 10.1016/j.neuron.2016.04.015 PMC4896746

[R5] Berton O, McClung CA, Dileone RJ, Krishnan V, Renthal W, Russo SJ, Graham D, Tsankova NM, Bolanos CA, Rios M, Monteggia LM, Self DW, Nestler EJ (2006) Essential role of BDNF in the mesolimbic dopamine pathway in social defeat stress. Science 311(5762): 864–8. 16469931 10.1126/science.1120972

[R6] Calhoon GG, and Tye KM (2015) Resolving the neural circuits of anxiety. Nat Neurosci. 2015 Oct;18(10):1394–404. DOI: 10.1038/nn.4101 26404714 PMC7575249

[R7] Cao J, Covington HE, Friedman AK, Wilkinson MB, Walsh JJ, Cooper DC, Nestler EJ, Han M (2010) Mesolimbic Dopamine Neurons in the Brain Reward Circuit Mediate Susceptibility to Social Defeat and Antidepressant Action. J. Neurosci. 30 (49): 16453–16458. 21147984 10.1523/JNEUROSCI.3177-10.2010 PMC3061337

[R8] Charney DS, Manji HK (2004) Life stress, genes, and depression: multiple pathways lead to increased risk and new opportunities for intervention. Sci STKE 225: re5. 10.1126/stke.2252004re5 15039492

[R9] Chou D, Huang CC, Hsu K (2014) Brain-derived neurotrophic factor in the amygdala mediates susceptibility to fear conditioning. Exp Neurol. 255: 19–29. 24582917 10.1016/j.expneurol.2014.02.016

[R10] Craske M.G., Treanor M., Conway C.C., Zbozinek T., and Vervliet B. (2014) Maximizing exposure therapy: An inhibitory learning approach. Behav Res and Ther 58, 10–23. 24864005 10.1016/j.brat.2014.04.006 PMC4114726

[R11] Christoffel DJ, Golden SA, Dumitriu D, Robison AJ, Janssen WG, Ahn HF, Krishnan V, Reyes CM, Han MH, Ables JL, Eisch AJ, Dietz DM, Ferguson D, Neve RL, Greengard P, Kim Y, Morrison JH, Russo SJ (2011) IκB kinase regulates social defeat stress-induced synaptic and behavioral plasticity. J Neurosci. 31(1): 314–21. 21209217 10.1523/JNEUROSCI.4763-10.2011 PMC3219041

[R12] Dalton GL, Chuan D, Yu T, Wang YT, Florescobc SB, Phillips AG (2012) NMDA GluN2A and GluN2B receptors play separate roles in the induction of LTP and LTD in the amygdala and in the acquisition and extinction of conditioned fear. Neuropharmacology 62 (2): 797–806. 21925518 10.1016/j.neuropharm.2011.09.001

[R13] Deaconfirm (2006) Assessing nest building in mice. Nat Protoc. 1(3): 1117–9. 17406392 10.1038/nprot.2006.170

[R14] Erlich JC, Bush DE, Ledoux JE. (2012) The role of the lateral amygdala in the retrieval and maintenance of fear-memories formed by repeated probabilistic reinforcement. Front Behav Neurosci. 2012 Apr 10;6:16. doi: 10.3389/fnbeh.2012.00016. eCollection 2012. 22514524 PMC3322351

[R15] Furini C, Myskiw J, Izquierdo I (2014) The learning of fear extinction. Neurosci Biobehav Rev 47: 670–83. 25452113 10.1016/j.neubiorev.2014.10.016

[R16] Gaskill BN, Karas AZ, Garner J., Pritchett-Corning KR (2013) Nest building as an indicator of health and welfare in laboratory mice. J Vis Exp. 82: 51012. 10.3791/51012 PMC4108067 24429701

[R17] Golden SA, Covington HE, Berton O, Russo SJ (2011) A standardized protocol for repeated social defeat stress in mice. Nat Protoc. 6(8): 1183–1191. 21799487 10.1038/nprot.2011.361 PMC3220278

[R18] Grillon C. (2008) Models and mechanisms of anxiety: evidence from startle studies Psychopharmacology 199 421–437 18058089 10.1007/s00213-007-1019-1 PMC2711770

[R19] Hascoët M, Bourin M, and Nic Dhonnchadha B.A (2001) The mouse light-dark paradigm: a review. Prog Neuropsychopharmacol Biol Psychiatry 25: 141–166. 10.1016/s0278-5846(00)00151-2 11263750

[R20] Herry C, Ciocchi S, Demou SV, Müller C, and Lüthi A (2008) Switching on and off fear by distinct neuronal circuits. Nature 454: 600–606. 18615015 10.1038/nature07166

[R21] Hsieh L. S., Wen J. H., Miyares L., Lombroso P. J., & Bordey A. (2017). Outbred CD1 mice are as suitable as inbred C57BL/6J mice in performing social tasks. Neuroscience letters, 637, 142–147. 10.1016/j.neulet.2016.11.035 27871995 PMC5203811

[R22] Hultman R, Mague SD, Li Q, Katz BM, Michel N, Lin L, Wang J, David LK, Blount C, Chandy R, Carlson D, Ulrich K, Carin L, Dunson D, Kumar S, Deisseroth K, Moore SD, Dzirasa K (2016) Dysregulation of prefrontal cortex-mediated slow-evolving limbic dynamics drives stress-induced emotional pathology. Neuron 91: 439–452. 27346529 10.1016/j.neuron.2016.05.038 PMC4986697

[R23] Hultman R, Ulrich K, Sachs BD, Blount C, Carlson DE, Ndubuizu N, Bagot RC, Parise EM, Vu MT, Gallagher NM, Wang J, Silva AJ, Deisseroth K, Mague SD, MaCaron MG, Nestler EJ, Carin L, and Dzirasa K (2018) Brain-wide Electrical Spatiotemporal Dynamics Encode Depression Vulnerability. Cell 173: 166–180. 29502969 10.1016/j.cell.2018.02.012 PMC6005365

[R24] Jansow AM, Cooper MA, Huhman KL (2004) N-methyl-d-aspartate receptors in the amygdala are necessary for the acquisition and expression of conditioned defeat. Neuroscience 123 (3): 625–634. 14706775 10.1016/j.neuroscience.2003.10.015

[R25] Jasnow AM, Chanjun S, Jeris I, Michael D, Huhman KL (2005) Memory of social defeat is facilitated by cAMP response element-binding protein overexpression in the amygdala. Behavioral Neuroscience 119(4): 1125–1130. 16187840 10.1037/0735-7044.119.4.1125

[R26] Jiang B, Wang H, Wang JL, Wang YJ, Zhu Q, Wang CN, Song L, Gao TT, Wang Y, Meng GL, Wu F, Ling Y, Zhang W, Li JX (2019) Hippocampal Salt-Inducible Kinase 2 Plays a Role in Depression via the CREB-Regulated Transcription Coactivator 1-cAMP Response Element Binding-Brain-Derived Neurotrophic Factor Pathway. Biol Psychiatry 85(8): 650–666. 30503507 10.1016/j.biopsych.2018.10.004

[R27] Jiang L, Kundu S, Lederman JD, López-Hernández GY, Ballinger EC, Wang S, Talmage DA, Role LW (2016) Cholinergic signaling controls conditioned fear behaviors and enhances plasticity of corticalamygdala circuits. Neuron 90: 1057–70. 27161525 10.1016/j.neuron.2016.04.028 PMC4891303

[R28] Jirkof P (2014) Burrowing and nest building behavior as indicators of well-being in mice. J Neurosci Methods 234: 139–46. 24525328 10.1016/j.jneumeth.2014.02.001

[R29] Kessler RC, Petukhova M, Sampson NA, Zaslavsky AM, Wittchen HU (2012) Twelve-month and lifetime prevalence and lifetime morbid risk of anxiety and mood disorders in the United States. Int J Methods Psychiatr Res. 21(3):169–184. 22865617 10.1002/mpr.1359 PMC4005415

[R30] Mohamad Khalifeh, Hobeika Rouba, El Hayek Lauretta, Saad Joelle, Eid Fadi, El-Khoury Reine, Ghayad Litsa-Maria, (2020) Nicotine Induces Resilience to Chronic Social Defeat Stress in a Mouse Model of Water Pipe Tobacco Exposure by Activating BDNF Signaling. Behavioural Brain Research 382 (2020): 112499. 31978493 10.1016/j.bbr.2020.112499

[R31] Kim J, Lee S, Park K, Hong I, Song B, Son G, Park H, Kim WR, Park E, Choe HK, Kim H, Lee C, Sun W, Kim K, Shin KI, Choi S (2007) Amygdala depotentiation and fear extinction. PNAS 104(52): 20955–20960. 18165656 10.1073/pnas.0710548105 PMC2409248

[R32] Kutlu M. G., & Gould T. J. (2015). Nicotine modulation of fear memories and anxiety:10.1016/j.bcp.2015.07.029 PMC4600451 26231942

[R33] Lagace DC, Donovan MH, DeCarolis NA, Farnbauch LA, Malhotra S, Berton O, Nestler EJ, Krishnan V, Eisch AJ (2010) Adult hippocampal neurogenesis is functionally important for stress-induced social avoidance. PNAS 107 (9): 4436–4441. 20176946 10.1073/pnas.0910072107 PMC2840117

[R34] Letzkus JJ, Wolff SBE, Meyer EM, Tovote P, Courtin J, Herry C, Lüthi A (2011) A disinhibitory microcircuit for associative fear learning in the auditory cortex. Nature 480: 331–335. 22158104 10.1038/nature10674

[R35] Lissek S., Powersb A.S., McMlurea E.B., Phelosc E.A., Woldehawariata G., Frillona C., and Pin D.S. (2004). Classical fear conditioningin the anxiety disorders: a meta-analysis Behav Res and Ther 49 1391–1424. 10.1016/j.brat.2004.10.007 15885654

[R36] Mallei A, Ieraci A, Popoli M (2018) Chronic social defeat stress differentially regulates the expression of BDNF transcripts and epigenetic modifying enzymes in susceptible and resilient mice. The World Journal of Biological Psychiatry: ePub. 10.1080/15622975.2018.1500029 30058429

[R37] Maren S (2015) Out with the old and in with the new: Synaptic mechanisms of extinction in the amygdala. Brain Res 1621: 231–238. 25312830 10.1016/j.brainres.2014.10.010 PMC4394019

[R38] McEwen BS (2017) Neurobiological and Systemic Effects of Chronic Stress. Chronic Stress 1: 1–11. 10.1177/2470547017692328 PMC5573220 28856337

[R39] Mineur YS, Fote GM, Blakeman S, Cahuzac ELM, Newbold SA, and Picciotto MR (2016) Multiple nicotinic acetylcholine receptor subtypes in the mouse amygdala regulate affective behaviors and response to social stress. Neuropsychopharmacology 41: 1579–1587. 26471256 10.1038/npp.2015.316 PMC4832019

[R40] Mineur Yann S, and Picciotto Marina R. (2019) The Role of Acetylcholine in Negative Encoding Bias: Too Much of a Good Thing? European Journal of Neuroscience, 2019. 10.1111/ejn.14641 PMC7282966 31821620

[R41] Monkol L (2016) Analysis of protein-coding genetic variation in 60,706 humans. Nature 536: 285–291. 27535533 10.1038/nature19057 PMC5018207

[R42] Morgan MA, Romanski LM, and LeDoux JE (1993) Extinction of emotional learning: contribution of medial prefrontal cortex. Neurosci Lett. 163: 109–13. 8295722 10.1016/0304-3940(93)90241-c

[R43] Morishita H, Miwa JM, Heintz N, and Hensch TK (2010) Lynx1, a cholinergic brake limits plasticity in adult visual cortex: (a cure for amblyopia through nicotinic receptor signaling). Science 33: 1238–1240. 10.1126/science.1195320 PMC3387538 21071629

[R44] Noack J, Richter K, Laube G, Haghgoo HA, Veh RW, Engelmann M (2010) Different importance of the volatile and non-volatile fractions of an olfactory signature for individual social recognition in rats versus mice and short-term versus long-term memory. Neurobiol Learn Mem. 94(4): 568–75. 20888419 10.1016/j.nlm.2010.09.013

[R45] Pagliusi MOF, Bonet IJM, Dias EV, Vieira AS, Tambeli CH, Parada CA, Sartori CR (2018) Social defeat stress induces hyperalgesia and increases truncated BDNF isoforms in the nucleus accumbens regardless of the depressive-like behavior induction in mice. Eur J Neurosci: EPub, ejn. 13994. 10.1111/ejn.13994 29885271

[R46] Picciotto MR (2003) Nicotine as a modulator of behavior: beyond the inverted U. Trends Pharmacol Sci 24: 493–499. 12967775 10.1016/S0165-6147(03)00230-X

[R47] Richter K, Wolf G, and Engelmann M (2005) Social recognition memory requires two stages of protein synthesis in mice. Learn Mem. 12(4): 407–13. 16077019 10.1101/lm.97505 PMC1183259

[R48] Saha R, Knapp S, Chakraborty D, Horovitz O, Albrecht A, Kriebel M, Kaphzan H, Ehrlich I, Volkmer H, Richter-Levin G (2017) GABAergic synapses at the axon initial segment of basolateral amygdala projection neurons modulate fear extinction. Neuropsychopharmacology 42: 473–484. 27634356 10.1038/npp.2016.205 PMC5399240

[R49] Schneiderman N, Ironson G, and Siegel S (2005) Stress and health: psychological, behavioral, and biological determinants. Annual Review of Clinical Psychology 1:1, 607–628. 10.1146/annurev.clinpsy.1.102803.144141 PMC2568977 17716101

[R50] Takahashi A, Chung J-R, Zhang S, Zhang H, Grossman Y, Aleyasin H, Flanigan ME, Pfau ML, Menard C, Dumitriu D, Hodes GE, McEwen BS, Nestler EJ, Han M-H, and Russo SJ (2017) Establishment of a repeated social defeat stress model in female mice. Scientific Reports 7: 12838. 28993631 10.1038/s41598-017-12811-8 PMC5634448

[R51] Vaishnav K, Han M-H, Graham DL, Berton O, Renthal W, Russo SJ, LaPlant Q, Graham A, Lutter M, Lagace DC, Subroto G, Reister R, Tannous P, Green TA, Neve RL, Chakravarty S, Kumar A, Eisch AJ, Nestler EJ (2007) Molecular Adaptations Underlying Susceptibility and Resistance to Social Defeat in Brain Reward Regions. Cell 131(2): 391–404. 17956738 10.1016/j.cell.2007.09.018

[R52] Wook Koo Ja, Labonté Benoit, Engmann Olivia, Calipari Erin S., Juarez Barbara, Lorsch Zachary, Walsh Jessica J., (2016) Essential Role of Mesolimbic Brain-Derived Neurotrophic Factor in Chronic Social Stress–Induced Depressive Behaviors. Biological Psychiatry 80, no. 6 (2016): 469–478. 26858215 10.1016/j.biopsych.2015.12.009 PMC4909591

[R53] Zhu G, Briz V, Seinfeld J, Liu Y, Bi X, Baudry M (2017) Calpain-1 deletion impairs mGluR-dependentLTD and fear memory extinction. Sci Rep 7: 42788. 28202907 10.1038/srep42788 PMC5311935

